# Effect of a Higher-Protein Nut versus Higher-Carbohydrate Cereal Enriched Diet on the Gut Microbiomes of Chinese Participants with Overweight and Normoglycaemia or Prediabetes in the Tū Ora Study

**DOI:** 10.3390/nu16121971

**Published:** 2024-06-20

**Authors:** Saif Faraj, Ivana R. Sequeira-Bisson, Louise Lu, Jennifer L. Miles-Chan, Michael Hoggard, Daniel Barnett, Amber Parry-Strong, Meika Foster, Jeremy D. Krebs, Sally D. Poppitt, Michael W. Taylor, Akarsh Mathrani

**Affiliations:** 1 Human Nutrition Unit, University of Auckland, Auckland 1024, New Zealand; sfar315@aucklanduni.ac.nz (S.F.); i.sequeira@auckland.ac.nz (I.R.S.-B.); louise.lu@auckland.ac.nz (L.L.); j.miles-chan@auckland.ac.nz (J.L.M.-C.); s.poppitt@auckland.ac.nz (S.D.P.); 2 High-Value Nutrition National Science Challenge, Auckland 1023, New Zealand; amber.parry-strong@ccdhb.org.nz (A.P.-S.); meika@edibleresearch.co.nz (M.F.); jeremy.krebs@otago.ac.nz (J.D.K.); 3 School of Biological Sciences, University of Auckland, Auckland 1010, New Zealand; michael.hoggard@auckland.ac.nz; 4 Department of Statistics, University of Auckland, Auckland 1010, New Zealand; dbar344@aucklanduni.ac.nz; 5 Department of Medicine, University of Otago, Dunedin 9054, New Zealand; 6 Centre for Endocrine, Diabetes and Obesity Research (CEDOR), Te Whatu Ora, Capital and Coast Health, Wellington P.O. Box 7902, New Zealand; 7 Edible Research, Ohoka, Christchurch 7475, New Zealand

**Keywords:** diet intervention, almonds, peanuts, overweight, prediabetes, 16S rRNA gene amplicon sequencing, shotgun sequencing, gut microbiome, functional potential

## Abstract

Global increases in metabolic disorders such as type 2 diabetes (T2D), especially within Asian populations, highlight the need for novel approaches to dietary intervention. The Tū Ora study previously evaluated the effects on metabolic health of including a nut product into the diet of a New Zealand cohort of Chinese participants with overweight and normoglycaemia or prediabetes through a 12-week randomised, parallel-group clinical trial. In this current study, we compared the impact of this higher-protein nut bar (HP-NB) versus a higher-carbohydrate cereal bar (HC-CB) on the faecal microbiome by employing both 16S rRNA gene amplicon and shotgun metagenomic sequencing of pre- and post-intervention pairs from 84 participants. Despite the higher fibre, protein, and unsaturated fat content of nuts, there was little difference between dietary groups in gut microbiome composition or functional potential, with the bacterial phylum *Firmicutes* dominating irrespective of diet. The lack of observed change suggests the dietary impact of the bars may have been insufficient to affect the gut microbiome. Manipulating the interplay between the diet, microbiome, and metabolic health may require a more substantial and/or prolonged dietary perturbation to generate an impactful modification of the gut ecosystem and its functional potential to aid in T2D risk reduction.

## 1. Introduction

Over the past two centuries, rapid urbanisation and industrialisation have been accompanied by substantial dietary changes and a generally more sedentary lifestyle. These changes are particularly pronounced in developing countries, in parallel with the increased adoption of Western dietary patterns [1,2]. These tend to be rich in highly processed, energy-dense, low-cost foods in contrast to traditional diets comprising fruits, vegetables, whole grains, nuts, and legumes [3,4,5]. Consequently, metabolic disorders such as obesity and type 2 diabetes (T2D) have become increasingly prevalent and now present a substantial global health issue [6]. Overweight and obesity affect some 2.5 billion people worldwide [7], with 537 million in 2021 estimated to be living with T2D, and this is projected to increase to 783 million by 2045 [6].

Asian peoples are particularly at risk, with a greater prevalence of adverse metabolic health in Asia than in other developed regions of the world [8,9,10]. One explanation for this susceptibility to metabolic disease is the propensity among Asian populations to deposit fat in the abdominal region, both as visceral adipose tissue [11,12,13] and ectopic organ fat, even in individuals without overweight or obesity. In 2012, Thomas et al. [14] coined this distinct phenotype the TOFI (Thin on the Outside Fat on the Inside) profile and added support to the ‘adipose tissue overflow’ hypothesis previously proposed by Sniderman and colleagues [15]. This hypothesis purports that the subcutaneous fat storage compartment in Asians is inherently smaller compared to that of Europeans and that, under circumstances of excess energy intake, its capacity is quickly exhausted, and lipid ‘overflows’ into both central/visceral compartments and non-adipose organ sites. This concept has received support from several subsequent studies [16,17,18], with the comparison of ethnically diverse populations showing that an individual of Asian descent with a low body mass index (BMI) of >25 kg/m^2^ has the equivalent T2D risk of Europeans with a higher BMI of >30 kg/m^2^ [19]. The TOFI profile has also been compared with the symptoms manifested in partial lipodystrophy [20], as both phenotypes display a similar predisposition towards lipid deposition in critical organs such as the pancreas and liver, often leading to the onset of insulin resistance and T2D [20,21].

Tree nuts and peanuts (commonly termed ‘ground nuts’) are highly nutritious sources of dietary fibre, plant protein, and unsaturated fats [22]. Fibre-rich diets have been linked with a decreased risk of cardiovascular [23] and inflammatory diseases [24], certain cancers [25], kidney stones [26], and metabolic disorders such as obesity and T2D [23]. In conjunction with dietary fibre, nutritionally dense dried fruits also offer a variety of essential minerals and polyphenols [27], which have been shown to mitigate T2D risk [28,29]. Indeed, multiple studies, both RCTs and observational, have reported various tree nuts (including pistachios and almonds) and ground nuts (peanuts) to be associated with improved metabolic health, including improvements in T2D-associated outcomes such as hyperglycaemia, insulin resistance, low-grade inflammation, and oxidative stress [22,30,31,32,33,34,35,36]. Although legumes such as peanuts have been the focus of fewer investigations, they share a similar nutritional profile to tree nuts [37], with considerable evidence also supporting their T2D-protective effects [32,38]. Notwithstanding these previous findings, there remains a need for further research. Unexpectedly, a recent meta-analysis of eight observational studies failed to reveal a significant association between total nut (including peanuts and tree nuts) consumption and T2D-related parameters [39].

The human gut microbiome, comprising hundreds of bacterial species as well as fungi, protozoa, archaea, and bacteriophages, is critical in metabolising undigested dietary components and maintaining metabolic homeostasis [40,41]. Dependent on the host diet for nutrients and energy harvest, the gut microbiome is shaped by the dietary patterns to which it is exposed, with diet shifts often accompanied by changes in gut microbial community structure and function [42]. Furthermore, diet, the gut microbiome, and disease are tightly linked, with implications for diverse conditions including obesity, T2D, and inflammatory bowel disease [43,44]. Healthy, balanced diets tend to support beneficial bacteria, whereas unhealthy diets may promote harmful bacteria [43]. For example, microbial fermentation of dietary fibre in the gut benefits short-chain fatty acid (SCFA) biosynthesis [42]. Acetate, propionate, and butyrate are key SCFAs that exhibit anti-inflammatory effects on gut epithelial and immune cells [45,46], with butyrate, in particular, notable for its capacity to directly support the growth and differentiation of host colonocytes and enterocytes [47,48]. Butyrate production has also been negatively associated with T2D onset, with butyrate-producing bacteria diminished in the gut microbiotas of people with T2D [49,50,51]. In a randomised, cross-over trial examining adults with elevated fasting glucose, the consumption of 28 g/day of peanuts led to the enrichment of *Roseburia*, a known butyrate producer [49]. Metatranscriptomic analysis identified an increase in the expression of an aerobic carbo-monoxide dehydrogenase gene associated with butyrate production, supporting the reported capability of peanuts in modulating the microbiome towards increased butyrate production [49].

Considering the increased risk of T2D presented by the TOFI profile and the rapid growth of the T2D burden in Asian communities, more T2D-prevention studies targeted explicitly towards Asian populations are important. The recent Tū Ora clinical trials [52,53] conducted in our New Zealand clinic investigated the effects of a nut-based product, containing the recommended 28 g/day of mixed nuts [54], on T2D-related outcomes in an at-risk Chinese population. In these two randomised controlled trials (RCTs), although consumption of a higher-protein nut bar led to a significantly attenuated post-prandial glycaemic response when compared to an iso-energetic, higher-carbohydrate cereal-based bar [52], no longer-term benefits of the nut bar on fasting endpoints were evident [53]. Here, we applied 16S rRNA gene amplicon and shotgun metagenomic approaches to faecal samples from the trial by Sequeira-Bisson and colleagues [53] to identify any shifts in microbiome composition and/or functional potential accompanying the higher-protein nut bar diet.

## 2. Materials and Methods

The microbiome study described in this paper was one component of the 12-week randomised, open allocation parallel-group Tū Ora RCT. The trial recruited participants from residential communities in Auckland and Wellington, New Zealand. It focused on the efficacy of a mixed nut product as a prebiotic therapy for T2D prevention in Asian Chinese consumers with overweight and normoglycaemia or prediabetes. The inclusion and exclusion criteria, as well as details of fasting plasma glucose (FPG) measurements to assess (pre)diabetes status, and primary outcomes of the clinical trial have been published elsewhere [52,53]. All participants provided written informed consent, and ethics approval was obtained via the National Health and Disabilities Ethics Committee (HDEC), Auckland, New Zealand (18/NTB/1/). The trial was registered with the Australian New Zealand Clinical Trial Registry (Trial ID: ACTRN12618000476235).

**Figure 1:**
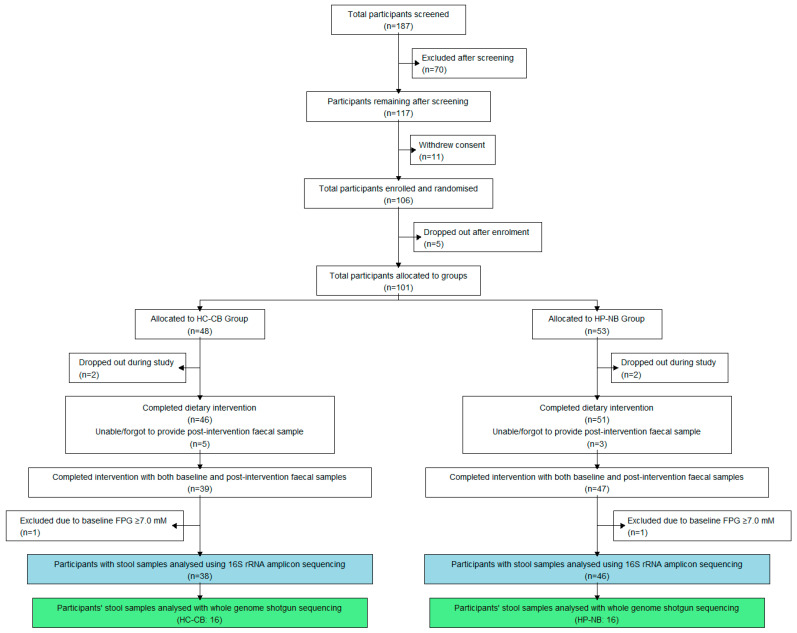
CONSORT participant flow chart for the microbiome component of the Tū Ora dietary intervention trial. This flow chart depicts the number of participants who undertook the 12-week intervention and provided faecal samples. Blue boxes indicate the number of samples analysed using 16S rRNA gene amplicon sequencing, while green boxes represent samples analysed using shotgun metagenomics. Participants were assigned to either the higher-protein nut bar (HP-NB) or higher-carbohydrate cereal bar (HC-CB) dietary supplement.

One hundred and six individuals were eligible after in-clinic screening and subsequently enrolled in the trial (Figure 1). Forty-eight participants were randomised into the high-carbohydrate cereal bar (HC-CB) group and 53 into the high-protein nut bar (HP-NB) group, with both dietary arms including participants with either normoglycaemia or prediabetes. Participant demographic data are presented in Table 1. Participants in the HC-CB cohort were provided with 1 MJ bars rich in cereal carbohydrates, and participants in the HP-NB cohort were provided with iso-energetic 1 MJ bars rich in nut protein, unsaturated fats, and fibre (for full details, see [52]). Participants were instructed to maintain all aspects of their habitual diet and activity levels for the 12-week trial period, except for the inclusion of one bar in their diet daily, at breakfast or as a snack. They were encouraged to substitute the bar for another food item to ensure they did not increase their total daily energy intake and, hence, not increase body weight.

### 2.1. Faecal Sample Collection

Faecal samples were self-collected by participants at baseline and after the 12-week dietary intervention and delivered to the research clinic. Participants were given a sample collection kit, including a kidney dish, a scoop, a container, and a small sterile collection tube. After defecation into the kidney dish and using the dedicated scoop to securely collect a portion of faecal matter into the sterile tube, participants placed the tube inside the provided container half-filled with water and stored the unit at −18 °C in their home freezer until delivery to the clinic. The outer ice layer prevented the thawing of samples during transit. Upon arrival at the laboratory, all faecal samples were stored at −80 °C before DNA extraction. Participants provided two faecal samples: one at clinical investigation day 1 (CID 1; baseline) and one at CID 5 (post-12-week intervention), representing a patient sample pair.

### 2.2. DNA Extraction

Genomic DNA was extracted from 250 mg of faecal sample using the International Human Microbiome Standards (IHMS) Protocol #9 [55], a repeated bead-beating method utilising 0.1 mm silica and 3 mm glass beads. Cell lysis was performed using a non-commercial lysis buffer (500 mM NaCl, 50 mM Tris-HCl at pH 8.0, 50 mM EDTA, and 4% SDS) as per the protocol; however, a Qiagen Tissuelyser II (Retsch, Haan, Germany) was used (frequency of 30 Hz, for two cycles of 1.5 min) to break the cells instead of the recommended FastPrep^®^-24 Instrument (MP Biomedicals, Irvine, CA, USA; 116004500). A QIAamp DNA Minikit (Qiagen, Venlo, The Netherlands; 51306) was utilised in the final steps of the protocol to remove RNA and protein, for purification, per the protocol. Negative DNA extractions containing 250 µL of sterile water instead of 250 mg faecal sample were also carried out to test for potential contamination. All extracts were subsequently analysed on a Nanodrop 3300 fluorospectrometer (Nanodrop Technologies Inc., Wilmington, DE, USA) to determine DNA quality and concentration.

### 2.3. 16S rRNA Gene-Targeted PCR and Sequencing

Bacterial community structure was analysed for all 168 samples (from 84 participants) by PCR amplification and sequencing of the V3–V4 region of the 16S rRNA gene. The KAPA High Fidelity HotStart Readymix PCR Kit (Kapa Biosystems^®^, Wilmington, MA, USA) was utilised, with 50 ng of template genomic DNA used per reaction. The 341F-785R primer pair [56], with added Illumina MiSeq-compatible adaptors (Illumina Inc., San Diego, CA, USA), was used with the following thermocycling conditions: initial denaturation and activation of enzymes at 95 °C for 3 min, followed by 25 cycles of denaturation (95 °C for 30 s), annealing (55 °C for 30 s), and elongation (72 °C for 30 s), with a final extension of 72 °C for 10 min. Correct amplicon size was confirmed by electrophoresis on 1% (*w*/*v*) agarose gels with SYBR Safe nucleic acid stain (Invitrogen Co., Carlsbad, CA USA). Negative PCR controls, in which nuclease-free H_2_O was used instead of template DNA and amplifications of eluates from the negative DNA extractions, did not produce any visible DNA products. Randomly selected negative controls were nonetheless sequenced even if no product was visible on an agarose gel. PCR amplicons were purified using AMPure magnetic beads (Beckman Coulter Inc., Brea, CA, USA) and quantified using the Qubit dsDNA high-sensitivity kit (Invitrogen Co., USA). DNA concentrations of the purified samples were standardised, and Auckland Genomics Ltd. (Auckland, New Zealand) carried out Illumina MiSeq sequencing (2 × 300 bp chemistry).

### 2.4. Shotgun Metagenome Sequencing

Samples from all participants with prediabetes were further interrogated using shotgun metagenome sequencing. This sub-analysis comprised 64 pre- and post-intervention sample pairs analysed from each of the 32 participants with prediabetes. DNA was extracted from faecal sample pairs via a repeated bead-beating method [55]. The extracted DNA was eluted using AE elution buffer (Qiagen, The Netherlands; 51306 DNA Minikit) and quantified with the Qubit dsDNA high-sensitivity kit (Invitrogen Co., USA). Quality was assessed as a measure of absorbance using a Nanodrop 3300 fluorospectrometer (Nanodrop Technologies Inc., Wilmington, NC, USA), and extract integrity was assessed through 0.7% agarose gel electrophoresis using a lambda HindIII ladder for comparison. Library preparation was conducted using Thruplex DNA libraries, and sequencing on HiSeq 2500 V4, using 2 × 125 bp paired-end reads, was conducted by Otago Genomics Ltd. (University of Otago, Dunedin, New Zealand).

### 2.5. Bioinformatic and Statistical Analysis of Sequence Datasets

Processing of the 16S rRNA gene amplicon dataset began with removing Illumina adaptors and primers using Trimmomatic under standard settings [57]. Cleaned reads were then processed into amplicon sequence variants (ASVs) using the DADA2 pipeline on the New Zealand eScience Infrastructure (NeSI) computing cluster [58]. Reads were trimmed in DADA2 at 280 and 200 bp for forward and reverse reads, respectively, aiming for a median quality score of >30. After merging paired reads, an ASV table was constructed, and chimaeras were removed [58]. Taxonomic assignment was performed at the genus level using the SILVA 138.1 ribosomal RNA database and the IdTaxa function from DECIPHER (version 3.18) [59]. DADA2 outputs were imported and assembled in R Studio (v 4.3.2) using the phyloseq package (Bioconductor version 3.18) [60]. Normalisation was performed to facilitate comparison across samples with different sampling depths: each sample was standardised to 6500 reads using the scaling with ranked subsampling method component of the SRS package [61], with the C-min set to ~6500 reads. No samples were discarded.

FastQ files provided by the sequencing facility were subjected to quality filtering and trimming using BBDuk for analysis of the shotgun metagenome dataset. This comprised the removal of (1) adapter sequences, (2) any retained PhiX sequences from reads, and (3) trimming 7–8 bp of poorer quality at the start and 2–3 bp at the end of reads. Post-QC sequences were then checked for over-filtering or over-trimming with FastQC. Human DNA was subsequently removed using BBTools from the BBMap suite to map reads against a specially processed human genome reference (hg19_main_mask_ribo_animal_allplant_allfungus.fa.gz) [62]. Resulting reads were aligned using DIAMOND Blast X against the NCBI-nr protein database for functional annotation [63]. Taxonomic and functional binning was then performed using the MEGAN 6 daa-meganizer tool with the megan-map-Feb2022 database, which computes taxonomic and functional classifications of all reads against several databases as recommended by the MEGAN 6 short reads pipeline using the LCA algorithm [63]. Finally, the meganized files were compared against each other using the compute-comparison tool, and the resultant outputs from each database (NCBI, KEGG) were exported from MEGAN 6 for assembling into a phyloseq object in R Studio (v 4.3.2). Cumulative scale normalisation was applied on the shotgun dataset using the metagenomeSeq package to produce a normalised object. The pipeline was performed using the New Zealand eScience Infrastructure (NeSI) computing cluster with subsequent phyloseq and analysis work being conducted locally in R Studio (v 4.3.2).

Bacterial community diversity indices for both (16S rRNA and shotgun metagenome) datasets were calculated in R Studio (v 4.3.2). Alpha-diversity indices (Observed, Shannon, Simpson) were calculated using the estimate_richness() function from the phyloseq package and visualised using plot_richness() [60]. Paired Wilcoxon signed-rank tests were applied to compare alpha-diversity measures between diet groups, and *p*-values were adjusted with Benjamini–Hochberg multiple corrections. Counts were aggregated at the phylum and genus levels using tax_glom() for subsequent Bray–Curtis dissimilarity analysis using distance() and PCoA analysis using plot_ordination() from the phyloseq package. PERMANOVA (permutational multivariate analysis of variance) was conducted on Bray–Curtis distances using adonis2() from the vegan package (version 2.6-4) [64], with the Bray–Curtis dissimilarity set as the response variable, and CID as the explanatory variable. This analysis accounts for the non-independence of samples from the same patient (repeated measures, paired samples) by specifying the patient as the stratification variable. The analysis was set to 1999 permutations to test if bacterial community composition does not differ by CID. This PERMANOVA was followed by a custom permutation, which was designed to run on a loop. Permuting the CID variable within the patient block and performing the PERMANOVA for each permutation, the resultant R^2^ value was recorded (variance explained by permuted CID). Then, the proportion of permuted R^2^ values equal to or larger than the original R^2^ was used to calculate the empirical *p*-value, assessing the significance between bacterial community composition and CID. The PERMANOVA + custom loop permutation was applied to both datasets by subsetting the data by Diet, Sex, Glycaemic state, and Diet × Glycaemic state interaction where appropriate. Dissection of the dataset in this way is integral to assessing the influence of CID on bacterial community composition across clinical and demographic strata.

To evaluate the effect of the dietary interventions on bacterial community composition, we calculated the Bray–Curtis dissimilarity for each participant’s sample pair at baseline (CID1) and post-intervention (CID5). These values were then compared to quantify the ‘dissimilarity change’, a numerical value representing the shift in bacterial community composition over the intervention period. Subsequently, a Wilcoxon rank-sum test was used to assess statistically significant dissimilarity changes between diet groups (HP-NB, HC-CB) in the taxonomic and functional data of the shotgun metagenomic dataset.

Top taxa by relative abundance for phylum and genus levels were sorted using top_taxa() function and plotted using ggplot2 (version 3.5.0), using RColorBrewer (version 1.1-3) for the colour schemes [65] for the generation of relative-abundance graphs.

## 3. Results

### 3.1. Participant Demographics at Baseline

Of the 97 participants who completed the dietary intervention, 86 provided a faecal sample at both baseline and 12 weeks. Two of these participants were later identified to have T2D (FPG > 7.0 mM at baseline), so they were excluded from further analysis, leaving a final set of 84 sample pairs (Figure 1). The normoglycaemic group contained 52 participants (HC-CB = 23; HP-NB = 29), and the prediabetes group had 32 participants (HC-CB = 15; HP-NB = 17) (Table 1). Although demographic and anthropometric variables such as mean participant age (47.1 years) and BMI (27.4 kg/m^2^) were well-balanced across cohort groups, the sex ratio was skewed towards females in the normoglycaemic group (17M:35F), with males overrepresented in the prediabetes group (21M:11F).

### 3.2. Taxonomic Composition of 16S rRNA Gene Amplicon Sequencing Data

**Figure 2:**
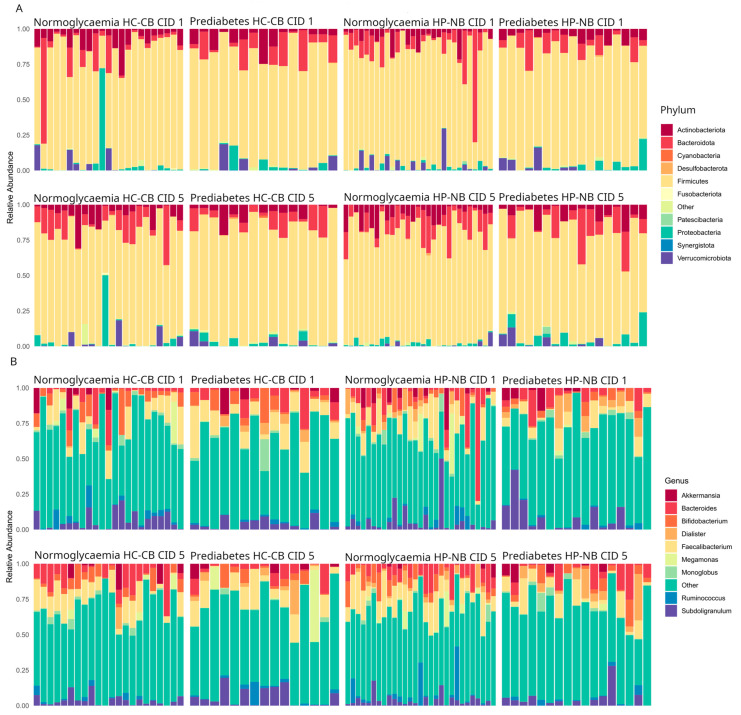
16S rRNA gene amplicon-based relative bacterial abundance in CID 1 (baseline) and CID 5 (post-intervention) samples. Relative abundances are shown for the 10 most abundant bacterial taxa at (**A**) phylum and (**B**) genus level. Data shown are for all 168 samples (i.e., CID 1 and CID 5 pairs from 84 participants). “Other” refers to less abundant taxa and those not classified at the respective taxonomic level. HP-NB, higher-protein nut bar; HC-CB, higher-carbohydrate cereal bar.

To determine microbiota composition, taxonomic profiles for the entire cohort (16S rRNA gene amplicon sequence data) were obtained for the 84 participants outlined above (Figure 2), with a total of 25,484 unique ASVs detected. Consistent with expectations for gut microbiome samples, the dominant bacterial phyla were *Firmicutes* (66.7% of total 16S rRNA gene sequences), *Bacteroidota* (16.6%), *Proteobacteria* (4.12%), and *Actinobacteriota* (4.10%), while *Verrucomicrobiota* (0.7%) were also relatively abundant. These patterns primarily held regardless of glycaemic status, diet group, or time point, though there was considerable variation among individuals (Figure 2A). At the genus level, *Faecalibacterium* (10%), *Bacteroides* (7.1%), *Subdoligranulum* (5.2%), *Bifidobacterium* (4.9%), and *Dialister* (2.6%) exhibited the highest relative abundances, although again, variation among individuals was substantial (Figure 2B).

### 3.3. Factors Affecting 16S rRNA Gene-Based Microbiota Composition

To isolate the effect of time (CID) per se, we sought to simplify the analysis by subsetting the 16S rRNA gene dataset by critical factors of interest, namely, sex, diet, and glycaemic status (Table 2). Of all tested comparisons, only when limiting the analysis to male participants was there a significant result (*p* = 0.017), and even then, only 1.2% of the observed variation in the microbiota was explained. PERMANOVA analysis of the shotgun metagenome dataset revealed a similar lack of statistically significant effects at both taxonomy (genus) and functional potential (KEGG) levels (Supplementary Table S5).

### 3.4. Taxonomic Composition and Functional Potential of the Faecal Microbiome Based on Shotgun Metagenomic Sequencing

For insights into the faecal microbiome’s functional potential and a PCR-independent examination of microbiota taxonomic composition, we employed shotgun metagenome sequencing for the 32 participants with prediabetes. A total of 1,063,123,328 sequence reads were obtained, with 1,042,409,216 (98%) of these able to be taxonomically assigned. Among these, *Firmicutes* (58.2% of total assigned sequence reads), *Bacteroidota* (21%), and *Proteobacteria* (2.8%) (Supplementary Figure S3) were the dominant bacterial phyla, while *Bacteroides* (6.7%), *Eubacterium* (1.3%), and *Subdoligranulum* (1.2%) were the most abundant genera (Supplementary Figure S4). Consistent with the 16S rRNA gene amplicon data, analysis of the shotgun dataset did not identify any significant differences in overall taxonomic composition due to diet. Alpha diversity (Shannon, Observed, Simpson metrics) did not differ significantly (Wilcoxon rank-sum test) with diet at either the genus level or KEGG level 2 (Supplementary Tables S3 and S4).

**Figure 3:**
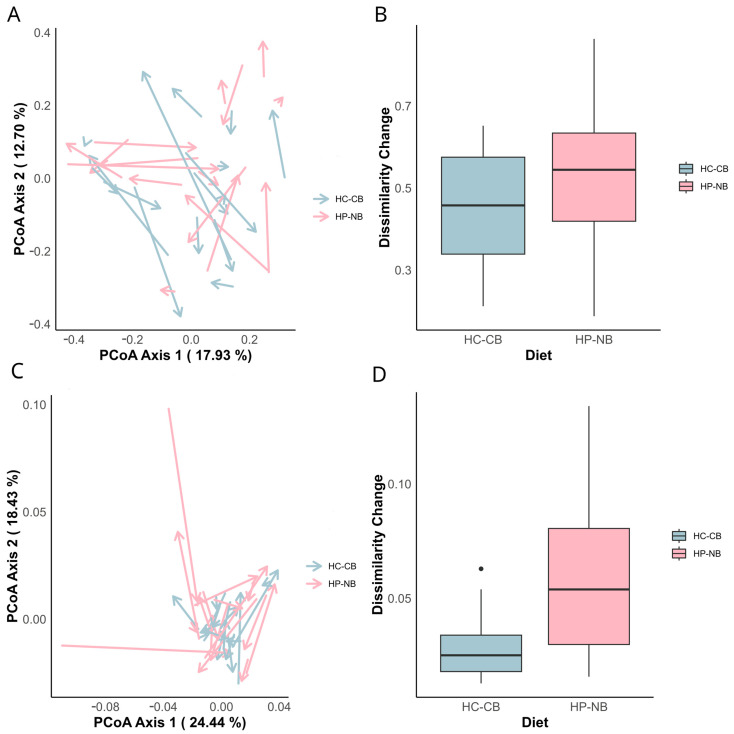
Shift in bacterial community composition and KEGG level 2 metabolic pathways (Bray–Curtis dissimilarity) for the shotgun metagenome dataset (restricted to participants with overweight and prediabetes, n = 32). (**A**,**C**) Principal coordinate analysis (PCoA) plots depict changes in the bacterial community (genus level) (**A**) and KEGG level 2 metabolic pathways (**C**) between CID 1 baseline and CID 5 end of intervention. Samples are colour coded by diet group (HP-NB: red; HC-CB: blue). Arrows represent the direction and magnitude of change in the bacterial community from CID 1 to CID 5. PERMANOVA analysis revealed no statistical significance by CID at the genus level (HP-NB *p* = 0.89; HC-CB *p* = 0.44) or at KEGG level 2 (HP-NB *p* = 0.31; HC-CB *p* = 0.998). (**B**,**D**) Box and whisker plots showing the dissimilarity change (Bray–Curtis dissimilarity) between CID 1 and CID 5 for each diet group: (**B**) dissimilarity change in the bacterial community (genus level); (**D**) dissimilarity change in KEGG level 2 metabolic pathways. Outliers are represented as individual dots.

While shifts in both bacterial community composition (Figure 3A,B) and functional potential (Figure 3C,D) were observed, neither the magnitude nor trajectory of these changes were consistent. Thus, genus-level taxonomic composition did not differ significantly between diet groups, though functional pathways defined at KEGG level 2 did (*p* = 0.011).

Further examination using PCoA at KEGG level 2 (Figure 3B) mirrored the taxonomic findings, indicating the lack of divergence in potential microbial function that could be attributed to CID within each diet group. However, there was a significant change in dissimilarity between diet groups, with the Wilcoxon rank-sum test generating *p* = 0.011 for KEGG level 2 (Figure 3D), with similar outcomes for KEGG levels 3 (*p* = 0.018), 4 (*p* = 0.020), and 5 (*p* = 0.020). These results suggest that diet type affected microbial functional potential.

**Figure 4:**
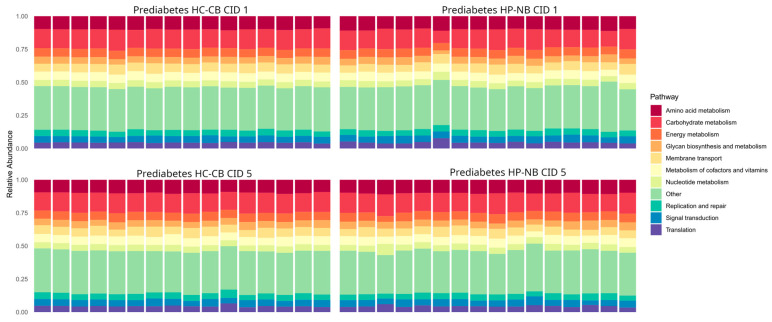
Relative abundance of KEGG (Kyoto Encyclopedia of Genes and Genomes) level 2 metabolic pathways. Shotgun metagenomic dataset restricted to participants with prediabetes (n = 32). CID, clinical investigation day; HP-NB, higher-protein nut bar; HC-CB, higher-carbohydrate cereal bar.

Like the taxonomic profile situation, the microbiome’s functional capacity did not differ between the HC-CB and HP-NB diets (Figure 4). At the read level, 566,866,944 were assigned to KEGG nodes. At KEGG level 2, the most abundant functional groups were carbohydrate metabolism, amino acid metabolism, and membrane transport, which were 9.22%, 6.44%, and 4.10%, respectively, with little or no variation between the diet groups.

## 4. Discussion

The Tū Ora dietary intervention trial investigated the effect of the recommended daily intake of nuts (28 g/day) on fasting and postprandial glycaemia and associated metabolic parameters in Chinese adults with overweight and normoglycaemia or prediabetes. The primary outcomes of this trial have been reported elsewhere, with significant improvements in postprandial [52] but not fasting glycaemic [53] endpoints. In our current analysis, we aimed to determine whether the daily inclusion of a 1 MJ higher-protein nut versus an iso-energetic higher-carbohydrate cereal product altered the composition and functional potential of the faecal microbiome among the Tū Ora participants. To this end, we employed a combination of 16S rRNA gene amplicon and shotgun metagenome sequencing.

Neither the gut microbiome’s composition nor its functional potential were significantly influenced by the inclusion of the 28 g/day nut product within the daily diet. Both 16S rRNA gene- and shotgun metagenome-based analyses indicated the dominance of the microbiome by members of the phylum *Firmicutes*, irrespective of diet (HP-NB, HC-CB) or glycaemic status (normoglycaemia, prediabetes), with *Bacteroidota* and—to a lesser extent—*Proteobacteria*, *Actinobacteriota*, and *Verrucomicrobiota* also exhibiting high relative abundance. At the genus level, the 16S rRNA gene amplicon analysis (conducted on all 84 participants, i.e., normoglycaemia and prediabetes cohorts) indicated the commonly reported gut bacteria *Faecalibacterium*, *Bacteroides*, and *Subdoligranulum* as having the highest relative abundance overall, with no evidence for significant differences in abundance across different diet groups. Though not entirely, this was mirrored by the shotgun metagenome data: in the prediabetes cohort (32 participants), *Bacteroides*, *Subdoligranulum*, and *Eubacterium* had the highest relative abundances. As is frequently seen in human microbiome studies, variation in the microbiome among individuals was substantial. Indeed, PERMANOVA analyses revealed that only a minor percentage of microbiome variation was explained by the tested factors (diet (HP-NB, HC-CB), glycaemic status (normoglycaemia, prediabetes), sex (male, female)), or the interaction between them. Much of the >95% residual variation unaccounted for by these factors is likely due to inherent inter-individual variability. Differences in habitual diet may also be important: wholesale diet changes—which would more likely heavily affect the microbiome—were not part of the Tū Ora trial, with the respective nut or cereal bar replacing only one small meal or snack per day.

Specific gut bacteria have previously been identified as key players in host metabolic health. Members of the genus *Faecalibacterium* (phylum *Firmicutes*) are butyrate producers with beneficial effects on gut health and anti-inflammatory properties [66]. *Bacteroides* (*Bacteroidota*) and *Subdoligranulum* (*Firmicutes*), both prominent in our findings, contribute to host digestion by fermentation of complex undigested carbohydrates and production of SCFAs as a by-product. SCFAs are important for gut health and contribute ~10% of human energy requirements [67,68]. *Akkermansia muciniphila*, a member of the phylum *Verrucomicrobiota*, is associated with positive health outcomes linked to its potential roles in maintaining gut-barrier integrity and exerting a protective effect against obesity and T2D [69]. The co-occurrence of these genera suggests the presence of a complex dynamic between members of the gut microbiome and host metabolism, emphasising their potential role in maintaining metabolic health.

The obtained shotgun metagenome data provide insights into the functional potential of the faecal microbiome, at least for those participants with overweight and prediabetes. Consistent with the taxonomy-based outcomes, in which we were unable to detect a significant effect of diet on the composition of the gut microbiome, the shotgun analyses did not reveal any significant effects on overall functional potential. Irrespective of KEGG level, functional potential varied little with diet group. Moreover, applying multiple differential abundance approaches failed to identify consistent metabolic pathways that were over- or under-represented in a specific diet group. The apparent lack of change in overall functional potential could reflect functional redundancy among microbiome members and/or insufficient microbiome perturbation by the imposed dietary interventions. Indeed, a recent 12-month intervention study highlighted the inherent resilience of gut microbiota despite changes in both diet and body weight [64].

Previous studies investigating the influence of nuts on the gut microbiome have varied somewhat in their outcomes, highlighting both the complex interplay between diet and gut health but also likely reflecting differences in study methodology, nut dosage, and nut type. A 2014 study [70] demonstrated a much stronger effect of pistachios compared with almonds on the gut microbiome, with notable enrichment of butyrate-producing bacteria. Butyrate producers *Roseburia* and *Faecalibacterium* were also among those bacteria which increased in abundance following the consumption of walnuts in various serving sizes [71]. It is thus likely that at least some nuts may act as a prebiotic, facilitating the growth and activities of SCFA producers [72]. The consumption of 28 g of peanuts daily enriched the proportion of butyrate-producing bacteria such as *Roseburia* and *Ruminococcaceae* while also increasing the expression of a butyrate-related gene in adults with elevated fasting glucose, emphasizing the potential use of peanuts as a gut microbiome modulator. Consumption of the Baru nut, an almond native to the Brazilian Cerrado biome, by individuals with obesity improved lipid profiles and was associated with changes in the gut microbiota [73]. Taken together, the available data indicate that nut consumption, including peanuts [49], can impact the gut microbiome in potentially positive ways, though this was not observed in our current findings from the Tū Ora trial.

There are several methodological considerations and limitations related to this study. The Tū Ora trial initially aimed to improve the glycaemic status in participants with obesity and prediabetes and recruited accordingly. However, a substantial proportion (roughly half) of participants reverted to normoglycaemia between initial screening and commencement of the intervention at CID 1. This reversion constrained the sample size and thus statistical power, albeit with the unexpected addition of a normoglycaemic cohort to our study. An absence of gene expression data further constrained our functional analysis, with our strategy for faecal collection and storage not lending itself to the preservation and analysis of RNA. Finally, and as alluded to earlier, our results may also be limited due to insufficient perturbation to the microbiome by the dietary intervention and the moderate duration (12-week) intervention period. Although largely consistent with previous studies, and certainly adhering to the recommended daily intake of nuts (28 g/day) [54], the intake of almonds and nuts in HP-NB may not have been adequate to elicit measurable changes in microbiome composition or function.

## 5. Conclusions

In conclusion, our study contributes to the body of knowledge around the use of nut-based dietary interventions in individuals with overweight and prediabetes to prevent the progression to T2D. The lack of significant influence of the applied intervention on microbiome composition or function may suggest a need to re-evaluate intervention parameters regarding nut dosage (>28 g/day) and dietary intervention period. Future research should consider higher dosages and more extended intervention periods to explore the potential for beneficial metabolic effects, while RNA-based methodologies to document microbial gene expression could also be insightful. Advancing our understanding of dietary interventions as a prediabetes management tool requires the further consideration of methodological aspects and dose size to better design practical strategies for T2D prevention in at-risk cohorts. Our study lays the groundwork for future investigation and supports the need for highly tailored, personalised dietary interventions to ameliorate and manage this global health challenge.

## Figures and Tables

**Table 1:** Baseline characteristics (at clinical investigation day 1, CID 1) of all participants from whom 16S rRNA gene amplicon sequencing data were obtained.

Variables	All	Diet		*p*-Value	Glycaemia		*p*-Value
		HP-NB	HC-CB		Normoglycaemia	Prediabetes	
n	84	38	46		52	32	
Age (y)	47.11, 11.04	46.81, 10.44	47.35, 11.93	0.83	45.63, 10.92	49.51, 11.06	0.12
Sex (M:F)	(38:46)	(22:24)	(14:24)	0.19	(17:35)	(21:11)	**<0.0001**
Height (m)	1.66, 0.86	1.65, 0.09	1.67, 0.07	0.22	1.66, 0.09	1.67, 0.07	0.61
Body weight (kg)	76.3, 14.6	73.6, 16.3	78.4, 11.3	0.11	75.3, 14.4	77.8, 14.5	0.46
BMI (kg/m^2^)	27.4, 3.63	27.0, 3.74	27.8, 3.27	0.3	27.2, 3.27	27.8, 3.98	0.46
Waist circumf. (cm)	91.9, 10.9	92.8, 11.3	90.8, 10.2	0.38	89.6, 9.3	95.6, 11.7	**0.018**
Hip circumf. (cm)	102.9, 8.2	103.8, 8.6	101.9, 7.8	0.27	102.7, 7.5	103.3, 9.0	0.77
FPG (mmol/L)	5.51, 0.50	5.47, 0.56	5.54, 0.43	0.48	5.21, 0.28	6.00, 0.39	**<0.0001**
Insulin (μU/mL)	12.48, 9.29	12.12, 8.37	12.91, 10.24	0.69	9.87, 9.85	16.71, 11.30	**0.0015**

Mean ± S.D., *p* < 0.05 independent sample *t*-test comparison. Data are presented for the entire cohort (All) and diet/glycaemia subgroups. HP-NB, higher-protein nut bar; HC-CB, higher-carbohydrate cereal bar; BMI, body mass index; circumf., circumference; FPG, fasting plasma glucose; Fisher’s *t*-test was used to test the categorical variable Sex. Significant *p*-values (*p* < 0.05) are in bold type.

**Table 2:** PERMANOVA results for Bray–Curtis dissimilarity by CID (accounting for repeated measures) applied on overall 16S rRNA gene amplicon sequencing data and subsets.

		Sex		Diet		Glycaemic Status		Diet × Glycaemic Status			
								HP-NB		HC-CB	
	All	Male	Female	HP-NB	HC-CB	N	P	N	P	N	P
n	84	38	46	38	46	52	32	23	15	29	17
R^2^	0.004	0.012	0.007	0.007	0.007	0.008	0.007	0.012	0.014	0.019	0.021
*p*-value	0.447	**0.017**	0.73	0.31	0.821	0.107	0.995	0.471	0.903	0.077	0.548

Subsets were defined based on three factors: sex (male, female), diet (HP-NB, higher-protein nut bar; HC-CB, higher-carbohydrate cereal bar), and glycaemic status (N, normoglycaemia; P, prediabetes). The table presents the results for all possible combinations of factors, including overall effect, effect within each sex level, diet level, and glycaemia level, and effect within each glycaemia level when stratified by diet. Significant *p*-values (*p* < 0.05) are shown in bold type.

## Data Availability

The 16S rRNA gene sequence data were deposited in Genbank under SRA Bioproject PRJNA900794 and the shotgun metagenome data under SRA Bioproject PRJNA1096331.

## Supplementary Materials

The following supporting materials can be downloaded at https://www.mdpi.com/article/10.3390/nu16121971/s1: Table S1: Baseline characteristics at clinical investigation day 1 (CID 1) of all participants from whom shotgun metagenomic sequencing data was obtained (n = 32); Figure S1: Shannon alpha-diversity index comparison from 16S rRNA gene sequencing (n = 84) across the normoglycemic and prediabetic cohorts at baseline (CID 1) and post-intervention (CID 5); Table S2: Paired Ranked Wilcoxon test results for alpha diversity indices of 16S rRNA gene-based sequencing dataset (n = 84) comparing alpha diversity at CID 1 (baseline) against CID 5 (post-intervention) at the genus level; Figure S2: Shotgun metagenomic sequencing-based relative abundance in CID 1 (baseline) and CID 5 (post-intervention) samples. Relative abundance is shown for the top 10 most abundant bacterial taxa at the phylum level; Figure S3: Shotgun metagenomic sequencing-based relative abundance in CID 1 (baseline) and CID 5 (post-intervention) samples. Relative abundance is shown for the top 10 most abundant bacterial taxa at the genus level; Figure S3: Shotgun metagenomic sequencing-based relative abundance in CID 1 (baseline) and CID 5 (post-intervention) samples. Relative abundance is shown for the top 10 most abundant bacterial taxa at the genus level; Figure S4: Shannon alpha-diversity index comparison from shotgun metagenomic sequencing (n = 32) across the prediabetic cohort at baseline (CID 1) and post-intervention (CID 5); Table S3: Paired Ranked Wilcoxon test results for alpha diversity indices of shotgun metagenomic sequencing dataset (n = 32) comparing alpha diversity at genus level at CID 1 (baseline) against CID 5 (post-intervention); Table S4: Paired Ranked Wilcoxon test results for alpha diversity indices of shotgun metagenomic sequencing dataset (n = 32) comparing alpha diversity at KEGG (Kyoto Encyclopedia of Genes and Genomes) level 2 at CID 1 (baseline) against CID 5 (post-intervention); Table S5: PERMANOVA results for Bray-Curtis dissimilarity by CID (accounting for repeated measures) applied on shotgun metagenome sequencing data (genus and KEGG levels) and subsets by diet (n = 32).
