# Supplementary material for: Effect of a Higher-Protein Nut versus Higher-Carbohydrate Cereal Enriched Diet on the Gut Microbiomes of Chinese Participants with Overweight and Normoglycaemia or Prediabetes in the Tū Ora Study
Source: Nutrients. 2024 Jun 20;16(12):1971. doi: 10.3390/nu16121971 (PMC11206330; doi:10.3390/nu16121971)
Supplement: Supplementary file 1 [file nutrients-16-01971-s001.zip › nutrients-3060450-supplementary.pdf]

Supplemental material for:

Effect of a Higher-Protein Nut versus Higher-Carbohydrate Cereal Enriched Diet on the Gut Microbiomes of Chinese Participants with Overweight and Normoglycaemia or Prediabetes in the Tū Ora study

Saif Faraj, Ivana R. Sequeira-Bisson, Louise Lu, Jennifer L. Miles-Chan, Michael Hoggard, Daniel Barnett, Amber Parry-Strong, Meika Foster, Jeremy D. Krebs, Sally D. Poppitt, Michael. W. Taylor, Akarsh Mathrani

Supplementary Table S1: Baseline characteristics at clinical investigation day 1, CID 1 of all participants from whom shotgun metagenomic sequencing data was obtained.

		Diet		
Variables	All	HP-NB	HC-CB	p-value
n	32	16	16	
Age (y)	49.8, 11.2	51.1, 10.7	48.4, 11.8	0.489
Height (m)	1.70, 0.07	1.68, 0.08	1.66, 0.06	0.400
Body weight (kg)	77.6, 14.3	77.4, 15.5	77.7, 13.2	0.942
BMI (kg/m2)	27.8, 4.0	27.3, 3.8	28.3, 4.2	0.482
FPG (mmol/l)	6.0, 0.43	6.1, 0.47	6.0, 0.38	0.342
Sex (M:F)	(21:11)	(11:5)	(10:6)	1

Mean ± S.D., p<0.05 independent sample t-test comparison. Data are presented for the shotgun metagenome cohort (prediabetes) and diet subgroups (n = 32). HP-NB, higher protein nut bar; HC-CB, higher carbohydrate cereal bar; BMI, body mass index; FPG, fasting plasma glucose; Fisher's t-test was used to test the categorical variable Sex.

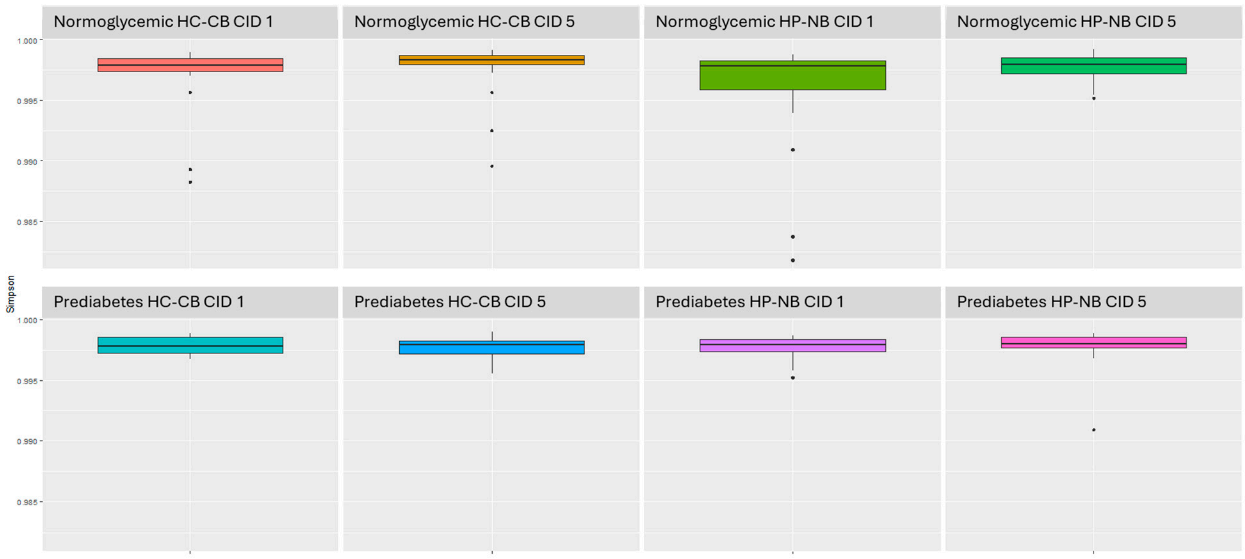

Supplementary Figure S1: Shannon alpha diversity index comparison from 16S rRNA gene sequencing (n = 84) across the normoglycemic and prediabetic cohorts at baseline (CID 1) and post-intervention (CID 5). HP-NB, higher protein nut bar; HC-CB, higher carbohydrate cereal bar. Outliers are represented as dots.

Supplementary Table S2: Paired Ranked Wilcoxon test results for alpha diversity indices of 16S rRNA gene-based sequencing dataset n = 84, comparing alpha diversity at CID 1 (baseline) against CID 5 (post intervention) at genus level p-values were adjusted with Benjamini Hochberg multiple corrections.

Alpha diversity index	Glycemic Status	Diet	Adjusted P value
Observed	Normoglycemic	HC-CB	0.235
	Normoglycemic	HP-NB	0.804
	Prediabetes	HC-CB	0.729
	Prediabetes	HP-NB	0.747
Shannon	Normoglycemic	HC-CB	0.105
	Normoglycemic	HP-NB	0.720
	Prediabetes	HC-CB	0.304
	Prediabetes	HP-NB	0.548
Simpson	Normoglycemic	HC-CB	0.075
	Normoglycemic	HP-NB	0.890
	Prediabetes	HC-CB	0.106
	Prediabetes	HP-NB	0.579

HP-NB, higher protein nut bar; HC-CB, higher carbohydrate cereal bar.

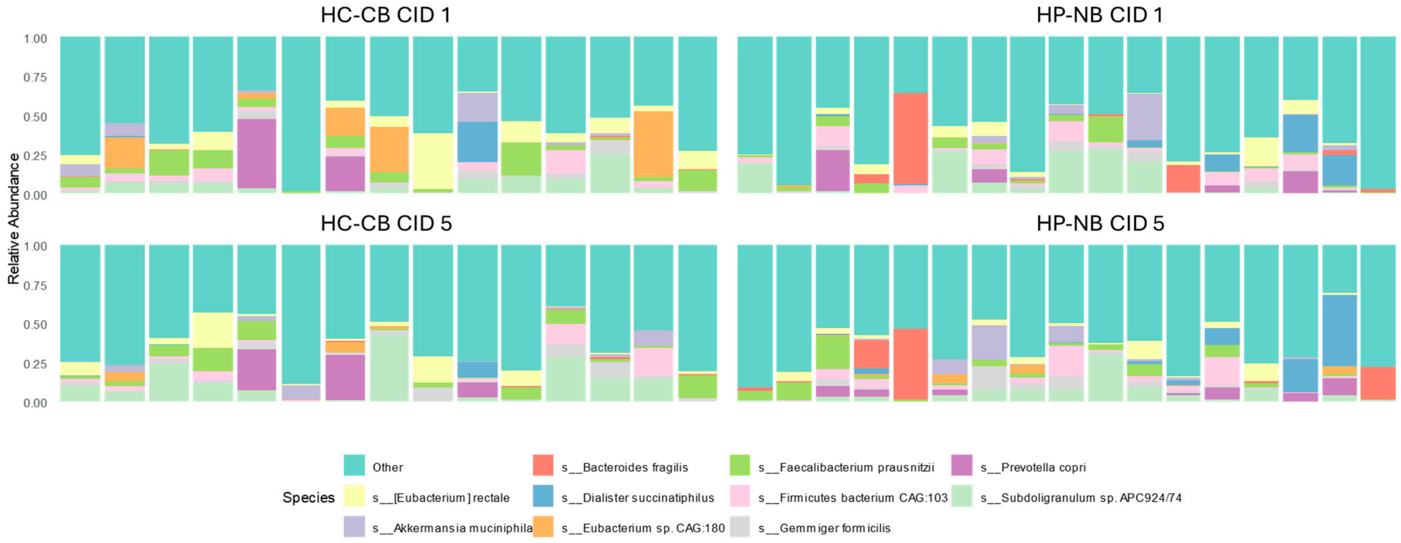

Supplementary Figure S2: Shotgun metagenomic sequencing based relative abundance in CID 1 (baseline) and CID 5 (post-intervention) samples. Relative abundance is shown for the top 10 most abundant bacterial taxa at phylum level. Data shown is for all 64 samples (i.e. CID 1 and CID 5 pairs from 32 participants). "Others" refer to less abundant taxa and those not classified. HP-NB, higher protein nut bar; HC-CB, higher carbohydrate cereal bar.

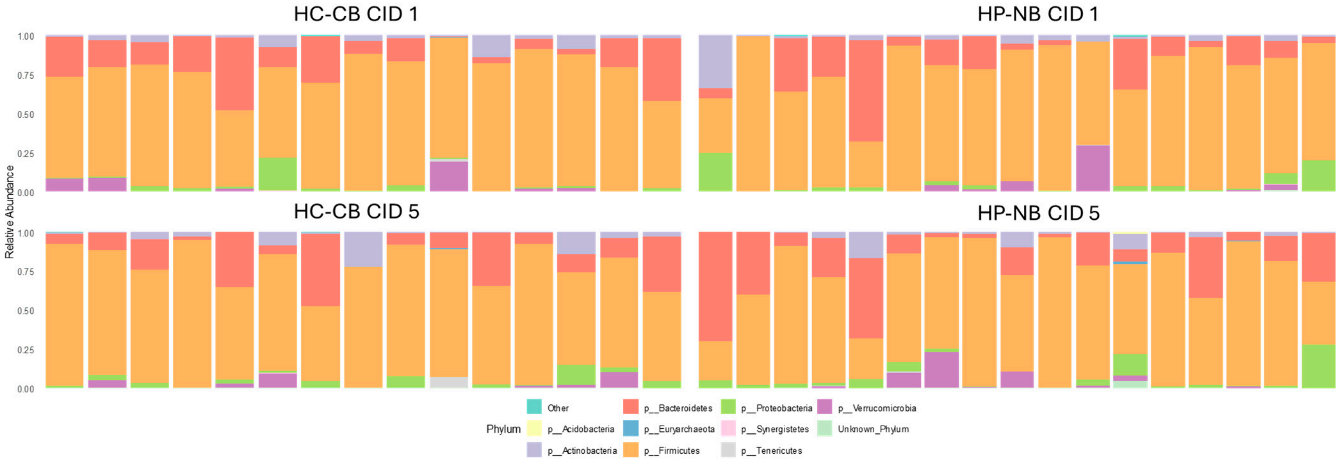

Supplementary Figure S3: Shotgun metagenomic sequencing based relative abundance in CID 1 (baseline) and CID 5 (post-intervention) samples. Relative abundance is shown for the top 10 most abundant bacterial taxa at genera level. Data shown is for all 64 samples (i.e. CID 1 and CID 5 pairs from 32 participants). “Others” refer to less abundant taxa and those not classified. HP-NB, higher protein nut bar; HC-CB, higher carbohydrate cereal bar.

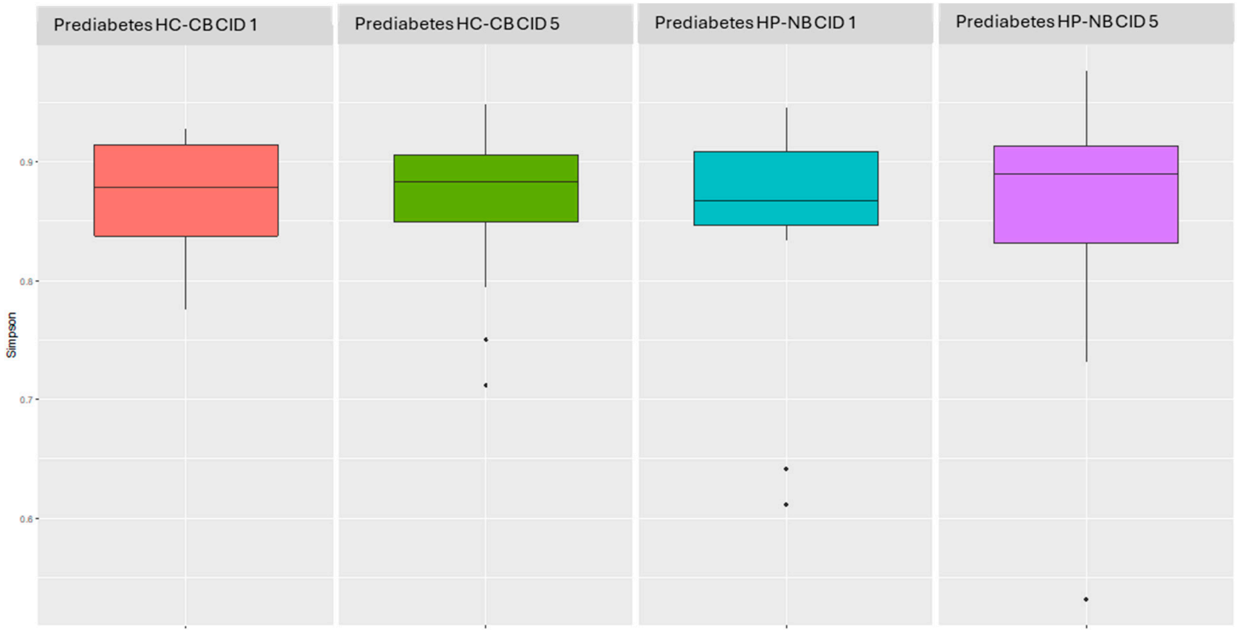

Supplementary Figure S4: Shannon alpha diversity index comparison from shot gut metagenomic sequencing (n = 32) across the prediabetic cohort at baseline (CID 1) and post-intervention (CID 5). HP-NB, higher protein nut bar; HC-CB, higher carbohydrate cereal bar. Outliers are represented as dots

Supplementary Table S3: Paired Ranked Wilcoxon test results for alpha diversity indices of shotgun metagenomic based sequencing dataset n = 32, comparing alpha diversity at genus level, at CID 1 (baseline) against CID 5 (post intervention) p-value were adjusted with Benjamini Hochberg multiple corrections. HP-NB, higher protein nut bar; HC-CB, higher carbohydrate cereal bar.

Measure	Diet	Adjusted p-value
Observed	HC-CB	0.304
Shannon	HC-CB	0.755
Simpson	HC-CB	0.977
Observed	HP-NB	0.981
Shannon	HP-NB	0.570
Simpson	HP-NB	0.636

Supplementary Table S4: Paired Ranked Wilcoxon test results for alpha diversity indices of shotgun metagenomic based sequencing dataset n = 32, comparing alpha diversity at KEGG (Kyoto Encyclopedia of Genes and Genomes) level 2, at CID 1 (baseline) against CID 5 (post intervention) with Benjamini Hochberg multiple corrections. HP-NB, higher protein nut bar; HC-CB, higher carbohydrate cereal bar.

Measure	Diet	Adjusted p-value
Observed	HC-CB	0.394
Shannon	HC-CB	0.485
Simpson	HC-CB	0.423
Observed	HP-NB	0.289
Shannon	HP-NB	0.245
Simpson	HP-NB	0.074

Supplementary Table S5: PERMANOVA results for Bray-Curtis dissimilarity by CID (accounting for repeated measures) applied on shotgun metagenome sequencing data (genus and KEGG levels) and subsets (by Diet) (n = 32). HP-NB, higher protein nut bar; HC-CB, higher carbohydrate cereal bar.

	Genus		KEGG Level 2		KEGG Level 3		KEGG Level 4		KEGG Level 5	
Diet	HC-CB	HP-NB	HC-CB	HP-NB	HC-CB	HP-NB	HC-CB	HP-NB	HC-CB	HP-NB
n					16					
R2	0.0165	0.0139	0.0029	0.0327	0.0139	0.0304	0.0178	0.0211	0.0178	0.0211
P-value	0.440	0.885	0.998	0.311	0.852	0.329	0.724	0.826	0.724	0.826
